# Measuring misclassification of Covid-19 as garbage codes: Results of investigating 1,365 deaths and implications for vital statistics in Brazil

**DOI:** 10.1371/journal.pgph.0000199

**Published:** 2022-05-05

**Authors:** Elisabeth B. França, Lenice H. Ishitani, Daisy Maria Xavier de Abreu, Renato Azeredo Teixeira, Paulo Roberto Lopes Corrêa, Eliene dos Santos de Jesus, Maria Antonieta Delgado Marinho, Tauá Vieira Bahia, Ana Luiza Bierrenbach, Philip Setel, Fatima Marinho

**Affiliations:** 1 Graduate Program in Public Health, School of Medicine, Federal University of Minas Gerais – Belo Horizonte, Minas Gerais, Brazil; 2 Research Group on Epidemiology and Health Evaluation, Federal University of Minas Gerais – Belo Horizonte, Minas Gerais, Brazil; 3 Municipal Health Department of Belo Horizonte, Belo Horizonte, Minas Gerais, Brazil; 4 Municipal Health Department of Salvador, Salvador, Bahia, Brazil; 5 National Health Foundation, Ministry of Health, Natal, Rio Grande do Norte, Brazil; 6 Prefeitura Municipal de Salvador, Salvador, Bahia, Brazil; 7 Vital Strategies, São Paulo, Brazil; 8 Vital Strategies, New York, New York, United States of America; APHRC: African Population and Health Research Center, KENYA

## Abstract

The purpose of this article is to quantify the amount of misclassification of the Coronavirus Disease-2019 (COVID-19) mortality occurring in hospitals and other health facilities in selected cities in Brazil, discuss potential factors contributing to this misclassification, and consider the implications for vital statistics. Hospital deaths assigned to causes classified as garbage code (GC) COVID-related cases (severe acute respiratory syndrome, pneumonia unspecified, sepsis, respiratory failure and ill-defined causes) were selected in three Brazilian state capitals. Data from medical charts and forensic reports were extracted from standard forms and analyzed by study physicians who re-assigned the underlying cause based on standardized criteria. Descriptive statistical analysis was performed and the potential impact in vital statistics in the country was also evaluated. Among 1,365 investigated deaths due to GC-COVID-related causes, COVID-19 was detected in 17.3% in the age group 0–59 years and 25.5% deaths in 60 years and over. These GCs rose substantially in 2020 in the country and were responsible for 211,611 registered deaths. Applying observed proportions by age, location and specific GC-COVID-related cause to national data, there would be an increase of 37,163 cases in the total of COVID-19 deaths, higher in the elderly. In conclusion, important undercount of deaths from COVID-19 among GC-COVID-related causes was detected in three selected capitals of Brazil. After extrapolating the study results for national GC-COVID-related deaths we infer that the burden of COVID-19 disease in Brazil in official vital statistics was probably under estimated by at least 18% in the country in 2020.

## Introduction

Monitoring deaths is an important element in evaluating mortality trends and vaccine effectiveness for COVID-19 [1]. However, there are many challenges in accurately determining the true level of mortality due to COVID-19. In Brazil, the most accurate data to assess cause-specific mortality are derived from the Ministry of Health Mortality Information System (SIM). For the identification of deaths from COVID-19 in this system, municipal health departments should actively search for death certificates in civil registry offices and also in hospitals and other health establishments. Each death certificate should be evaluated by the municipal surveillance team and subsequently coded and typed into the SIM. Cases with positive test results for the virus which cause COVID-19, from respiratory specimens, using real-time reverse transcription–polymerase chain reaction testing (reaction (RT-PCR) were classified as a laboratory-confirmed COVID-19 case [2].

Although the SIM has made significant progress in improving the accuracy of the national death certificate data over the past 10 years, being classified as of good quality by the Institute of Health Metrics and Evaluation (IHME) in 2010–2016 [3], the demands of the pandemic may have affected the accuracy of COVID-19 cause of death attribution in a few converging ways. For example, the timing of and diligence of proper completion of the medical certificate of cause of death, the international standardized death certificate, on which SIM data quality depends may have been affected. Physicians are being put to work with a large number of COVID-19 cases in an extremely stressful environment, leaving them with insufficient time to devote themselves in the task of carefully filling out of death certificates. In addition, many physicians are not well trained in correct certification practices, and errors in the cause of death attribution are common in Brazil [4,5]. Most importantly, restrictions on the provision of diagnostic tests in the country, which were particularly important during the onset of the epidemic [6], posed additional difficulties for physicians in correctly identifying deaths due to COVID-19.

Due to these issues, COVID-19 deaths may have been easily underestimated, as other causes of death were in previous years [7]. Although the classification of the cause of death by COVID-19 is subject to the same misclassification problems observed for other acute infectious diseases in the absence of confirmed laboratory diagnostic tests and/or other complementary exams, in 2020 there was an important increase in deaths due to ill-defined and other natural causes in Brazil [8,9]. The excess of deaths impacted life expectancy, and reduced the quality of classification of the underlying cause of death [10]. It should be observed that there was no other reason than the COVID-19 epidemic that could be pointed out as a reasonable explanation for the negative impact observed on the quality of mortality data in 2020, nor for the excess mortality.

Most misclassification, we hypothesize, would be as ill-defined causes or causes pertaining to the COVID-19 chain of events, i.e. codes that concern intermediate and immediate causes of death, such as severe acute respiratory syndrome (SARS), pneumonia unspecified, sepsis and respiratory failure [11]. These causes, when selected as underlying causes of death, are not sufficiently precise to be useful for public health intervention, or are simply implausible. Thus, they are categorized as garbage codes (GC) by the Global Burden of Disease-GBD study [3] (identified as ‘GC-COVID-related’ in this paper). Such misclassification could under-estimates the real magnitude of mortality due to the novel coronavirus.

In this study, we evaluate possible undercounts of COVID deaths through hospital and health facility investigation of GC-COVID-related deaths in three state capitals (Belo Horizonte, Natal and Salvador) during the initial first wave of the epidemic in Brazil in the first semester of 2020. We also evaluate the potential impact of this undercount on vital statistics for the country as a whole.

## Methods

### Data sources and procedures in hospital/health facility investigations

Hospital deaths with COVID-related garbage codes assigned as underlying causes of death were selected from municipal databases of three state capitals—Belo Horizonte, Salvador and Natal, the first two of them with more than 2 million inhabitants, from February to June 2020. The following codes from the International Classification of Diseases (ICD)-tenth Revision were included: sepsis (A40.0 to A41.9), unspecified pneumonia (J12.9, J15.9, J18), severe acute respiratory syndrome-SARS (J80, J98.8), acute and unspecified respiratory failure (J96.0 e J96.9), and ill-defined causes (R00.0-R94.9, R96, R98, R99). Some deaths in Salvador and Natal were selected before having been assigned an underlying cause of death by an official coder due to procedural delays in the release of the SIM database. These deaths were selected when COVID-19 appeared on any line of the death certificate and some of them (n = 256 deaths) were due to other underlying causes not GC-COVID-related. In Belo Horizonte, all selected deaths had already been coded with a COVID-related garbage code as the underlying cause of death.

Study assistants were trained to perform the field investigations. In the first stage of the investigation, they consulted two specific databases: the public health laboratory registry, in search of real-time polymerase chain reaction (RT-PCR) results for the diagnosis of coronavirus, and the SIVEP-Gripe registry, which is the surveillance database where all SARS cases and deaths should be compulsorily notified with their assigned etiologies and diagnostic methods. When the first stage of the investigation was inconclusive, the study assistants moved on to perform chart reviews in hospitals or emergency care units (UPAs), and also in the forensic institute in Salvador.

Data from medical charts and forensic reports were extracted to a standard form which has been used in the routine mortality surveillance investigation of deaths from ill-defined causes, named ‘hospital death investigation form for ill-defined causes’(IOCMD-H), with some small adaptations on a new form (IOCMD-COVID) introduced for improving detection of deaths from COVID-19. A single health professional in each city oversaw the data collection processes and supported the study assistants. Data collection was also carried out on reports from the death investigation service in Natal and in the forensic institute in Salvador.

### Data processing and analysis of investigated deaths

After the investigation process, IOCMD-COVID forms were entered into an online system, through which data were available to be reviewed by supervisors and the coordinator team. Feedback on inconsistencies and errors was provided to the local level. All on-line forms were analyzed by certifying study physicians who were previously trained in filling out the death certificate standardizing the post-investigation case classification process. Based on information collected in the IOCMD-COVID form (age, sex, date and place of death, date of symptom onset, signs and symptoms, history of contact with COVID-19 cases, examinations performed, medical care received, comorbidities and original causes of death defined by the attending physician), the certifying study physician re-assigned causes of death and the underlying cause, which were included in the on-line system.

Only one physician with broad experience in family health care was responsible for reviewing the data collected and certifying the cause of death in Belo Horizonte and Salvador. In Natal, two physicians worked together in the reassignment of causes. All physicians were previously trained in the completion of death certificates for the standardizing of post-investigation in the case classification process, and had had previous experience in this area. For each revised case, a justification was requested, with a degree of certainty of the diagnosis of the new underlying cause based on the following criteria:

1. COVID- definitive criterion: clinical picture compatible for the diagnosis of COVID-19 with the result of RT-PCR and/or IgM antibody and/or detectable antigen for SARS-Cov-2.
2. COVID- probable criterion: clinical picture compatible for the diagnosis of COVID-19 and with suggestive imaging (computed tomography of the lung) and/or epidemiological link, even with a single undetectable RT-PCR result.
3. COVID- possible criterion: clinical picture compatible with COVID in the absence of any supporting tests (specific or imaging), even with a single undetectable RT-PCR result.
4. Another specific diagnosis not COVID-19: criteria with degree of certainty as proposed previously [12].
5. Undetermined underlying cause: when the information obtained from hospital records did not allow for the identification of the degree of certainty of the diagnosis of specific diseases.

The degree of certainty in defining COVID-19 cases (categories 1, 2 and 3) or other specific causes (category 4) was determined by the certifying study physician when evaluating the forms. For cases in which the information obtained from hospital records did not permit the identification of a specific diagnosis, the original GC-COVID-related cause was maintained and the final cause was classified as undetermined underlying cause (category 5).

Descriptive statistical analysis was performed with simple and relative frequency of place of residence, age groups, original underlying cause and underlying cause after investigation. We also evaluated two periods of occurrence, February to April 2020 and May to June 2020. This was due to the dynamics of the epidemic and a substantial increase in cases after April (S1 Data).

#### Ethics statement

Our research project was approved by the Research Ethics Committee of the Federal University of Minas Gerais-UFMG under number 4155077. Data used in our research for analysis were fully anonymized and collected from the routine death investigation of the municipal mortality surveillance system. Because of this, the ethics committee waived the requirement for informed consent.

### Estimating the potential undercount of COVID-19 in the country in 2020

Firstly, we present the leading causes of death registered in the SIM in the country, comparing 2017 and 2020, according to the GBD 2017 list of causes of death [13], highlighting the garbage causes. For the codification of COVID-19 deaths, the Ministry of Health guidelines [14] established the use of ICD-10 code B34.2 with markers U07.1 (COVID-19, virus identified) and U07.2 (COVID-19, virus not identified. Secondly, we present the number of deaths registered as COVID-19 and as GC-COVID-related per month in 2020.

To estimate the potential undercount of COVID-19 in the country in 2020, we considered the proportions of each selected GC (SARS, pneumonia unspecified, sepsis, respiratory failure and ill-defined causes) reclassified as COVID-19 from February-June in the three municipalities, into age-groups 0–59 years and 60 years and over. We then applied these proportions to similar age-groups of SARS, pneumonia unspecified, sepsis, respiratory failure and ill-defined causes registered in 2020 in each of the 27 states of the country, redistributing as COVID-19 cases the same proportions detected in the selected cities. Estimated numbers of COVID-19 in states were then summed to calculate the national counts.

## Results

### Hospital/Health facility investigations in three selected municipalities

Among 1,819 registered deaths due to GC-COVID-related causes, we investigated 75%, with proportions varying from 84.5% in Salvador and 64% in Belo Horizonte and Natal (Table 1).

**Table 1:** Reported and investigated deaths in hospitals and health facilities according to selected causes and municipalities. February to June 2020.

Original cause of death	Belo Horizonte		
	Deaths reported	Deaths investigated	
		No.	%
SRAS	39	36	**92.3**
Unspecified pneumonia	287	182	**63.4**
Respiratory failure	7	2	**28.6**
Sepsis	95	63	**66.3**
Ill-defined causes	83	44	**53.0**
***GC-COVID-related***	**511**	***327***	**64.0**
	**Natal (ocurrence)**		
SRAS	52	49	**94.2**
Unspecified pneumonia	160	95	**59.4**
Respiratory failure	3	3	**100.0**
Sepsis	65	44	**67.7**
Ill-defined causes	49	20	**40.8**
***GC-COVID-related***	**329**	***211***	**64.1**
	**Salvador** *		
SRAS	149	140	**94.0**
Unspecified pneumonia	284	254	**89.4**
Respiratory failure	12	12	**100.0**
Sepsis	104	87	**83.7**
Ill-defined causes	430	334	**77.7**
***GC-COVID-related***	**979**	***827***	**84.5**
	**Total**		
SRAS	240	225	**93.8**
Unspecified pneumonia	731	531	**72.6**
Respiratory failure	22	17	**77.3**
Sepsis	264	194	**73.5**
Ill-defined causes	562	398	**70.8**
***GC-COVID-related***	**1819**	**1365**	**75.0**

Source: Mortality Information System;

* Includes deaths sent to the forensic institute (168).

After investigation, it emerged that 23.4% (n = 319) of GC-COVID-related deaths investigated from February-June 2020 in the selected municipalities were true COVID-19 cases (Table 2). The misclassification proportion varied by time-period. From February-April this proportion was 14.2%, but from May-June there was a greater COVID-19 confirmation among the GC-COVID-related causes (39.1%). Misclassification proportions also varied by the underlying cause originally reported in the death certificate, with the lowest proportion of misclassified COVID-19 detected among ill-defined causes (3.5%). Regarding the criterion used to confirm COVID, there was an increase from 9.0% to 44.4% in the definitive degree of COVID certainty (presence of laboratory evidence for COVID) from the first to the second period.

**Table 2:** Deaths from COVID-19 detected after investigation in selected municipalities according to confirmation criteria and period of occurrence. Brazil, February-June 2020.

Original cause of death	Deaths investigated	COVID-19 confirmed (total)		Confirmation criteria of COVID-19 cases						Other causes non-COVID-19			
				Definitive		Probable		Possible		Another diagnosis not related to COVID-19		Lack of information	
	n	n	%	n	%	n	%	n	%	n	%	n	%
***February-April***													
SARS	107	27	25.2	1	0.9	5	4.7	21	19.6	5	4.7	75	70.1
Unspecified pneumonia	345	82	23.8	10	2.9	16	4.6	56	16.2	8	2.3	255	73.9
Respiratory failure	9	3	33.3	0	0.0	1	11.1	2	22.2	1	11.1	5	55.6
Sepsis	137	8	5.8	0	0.0	1	0.7	7	5.1	4	2.9	125	91.2
Ill-defined causes	263	2	0.8	0	0.0	0	0.0	2	0.8	183	69.6	78	29.7
***GC-COVID-related***	**861**	**122**	**14.2**	**11**	**1.3**	**23**	**2.7**	**88**	**10.2**	**201**	**23.3**	**538**	**62.5**
***May-June***													
SARS	118	73	61.9	37	31.4	7	5.9	29	24.6	1	0.8	44	37.3
Unspecified pneumonia	186	90	48.4	39	21.0	10	5.4	41	22.0	1	0.5	95	51.1
Respiratory failure	8	6	75.0	2	25.0		0.0	4	50.0	0	0.0	2	25.0
Sepsis	57	16	28.1	8	14.0	1	1.8	7	12.3	2	3.5	39	68.4
Ill-defined causes	135	12	8.9	2	1.5	1	0.7	9	6.7	90	66.7	33	24.4
***GC-COVID-related***	**504**	**197**	**39.1**	**88**	**17.5**	**19**	**3.8**	**90**	**17.9**	**94**	**18.7**	**213**	**42.3**
**Total February-June**													
SARS	225	100	44.4	38	16.9	12	5.3	50	22.2	6	2.7	119	52.9
Unspecified pneumonia	531	172	32.4	49	9.2	26	4.9	97	18.3	9	1.7	350	65.9
Respiratory failure	17	9	52.9	2	11.8	1	5.9	6	35.3	1	5.9	7	41.2
Sepsis	194	24	12.4	8	4.1	2	1.0	14	7.2	6	3.1	164	84.5
Ill-defined causes	398	14	3.5	2	0.5	1	0.3	11	2.8	273	68.6	111	27.9
***GC-COVID-related***	**1,365**	**319**	**23.4**	**99**	**7.3**	**42**	**3.1**	**178**	**13.0**	**295**	**21.6**	**751**	**55.0**

Table 3 shows the investigations carried out and results stratified by age group. Among the 1,365 deaths investigated, pneumonia unspecified was the most frequently reported, particularly in the elderly. Approximately one third of pneumonia cases were due to COVID-19 in each age-group. Higher detection was seen for SARS and respiratory failure, but the latter had a low number of deaths investigated. On the other hand, sepsis and in particular ill-defined causes had the lowest proportions of cases reclassified as COVID-19. The COVID-19 detection among the total GC-COVID-related causes was 17.3% for the age group 0–59 years and higher among the elderly (25.5%).

**Table 3:** Deaths from COVID-19 detected after investigation in selected municipalities according to original cause of death and age-group. Brazil, February to June 2020.

Original cause of death	Investigated deaths	COVID confirmed	
	n	n	%
**0–59 years**			
SARS	49	27	55.1
Unspecified pneumonia	74	21	28.4
Respiratory failure	8	5	62.5
Sepsis	41	5	12.2
Ill-defined causes	181	3	1.7
***GC-COVID-related (total)***	***353***	***61***	**17.3**
**60 years and over**			
SARS	176	73	41.5
Unspecified pneumonia	457	151	33.0
Respiratory failure	9	4	44.4
Sepsis	153	19	12.4
Ill-defined causes	217	11	5.1
***GC-COVID-related (total)***	***1*,*012***	***258***	***25*.*5***
**All ages**			
SARS	225	100	44.4
Unspecified pneumonia	531	172	32.4
Respiratory failure	17	9	52.9
Sepsis	194	24	12.4
Ill-defined causes	398	14	3.5
***GC-COVID-related (total)***	**1,365**	**319**	***23*.*4***

Besides GC-COVID-related cases, we have also investigated 1,471 deaths originally labelled as suspected COVID-19 deaths (ICD-10 code B34.2 with the marker U07.2), i.e. without laboratory confirmation, before the entrance of these data in the official vital statistics of the selected municipalities. Among them, 1380 (93.8%) were confirmed as COVID-19 according to our standardized criteria.

Other underlying causes whose death certificates contained mention of COVID-19 were also investigated (n = 256 deaths), although not systematically. These other causes were mainly respiratory causes (57 cases), such as chronic obstructive pulmonary disease (COPD) and other pulmonary disorders, cardiovascular diseases (49), in particular cerebrovascular and ischemic heart disease, and neoplasms (44). COVID-19 was detected in a relatively high proportion (39.5% overall) among these causes not GC-COVID-related, ranging from 83.3% for COPD to 25.0% for neoplasms (S1 Table).

### Potential undercount of COVID-19 in the country in 2020

**Fig 1:**
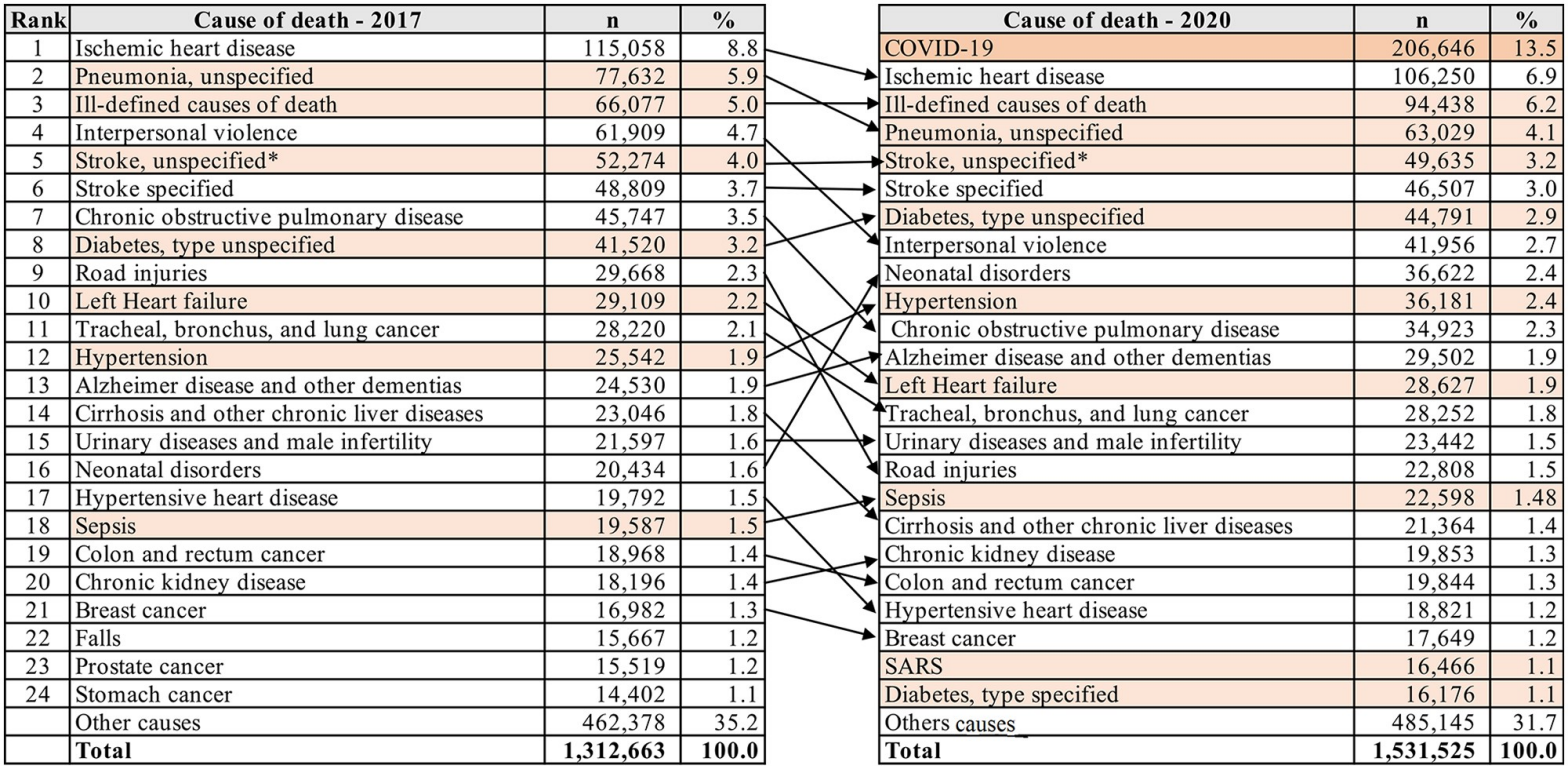
Leading causes of death in 2017 and 2020 in Brazil, with garbage codes highlighted. * code ICD-10 I67.8 not included; ** SARS in 2017: 3450 deaths (0.26% of total deaths) Source: Mortality Information System (SIM)- Accessed on April 23, 2021 (raw data).

Fig 1 highlights the impact in the country of the COVID-19 epidemic in the epidemiological profile in 2020 compared to 2017. Notably, COVID-19 was the first cause of mortality that contributed directly to almost 14% to the death total in Brazil in 2020. There was also an increase of garbage codes, in particular SARS and ill-defined causes which had the largest increase from 2017 to 2020. Increase in the rank of hypertension also occurred, from 12^th^ to 10^th^ position.

**Fig 2:**
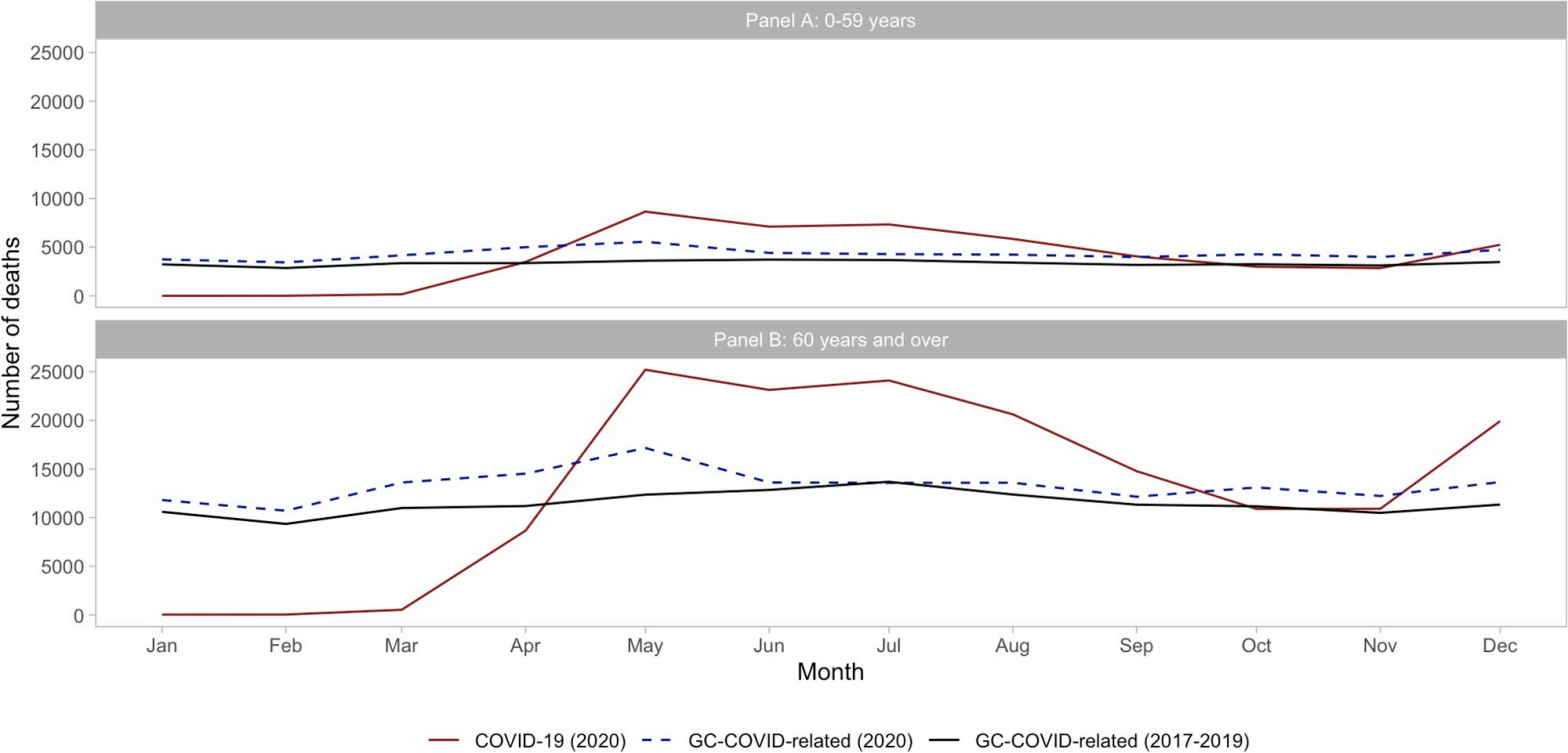
Number of COVID-19 and GC-COVID-related deaths in 2020 compared to the average number of GC-COVID-related deaths in 2017–2019, by months, among age-groups 0–59 years (Panel A) and 60 years and over (Panel B) in Brazil.

Fig 2 presents COVID-19 and GC-COVID-related by month in 2020. May-August had higher numbers of COVID-19 deaths and then after a downward trend. The second wave of COVID-19 started to take hold in Brazil in November. In both age groups, there is an excess of GC-COVID related deaths in 2020 compared with the average from 2017–2019. For the age 0–59 years, the expected number of COVID-related deaths was 3,600 in May and there was an excess of deaths of 54% in 2020, while for old ages it was lower (39%) (Fig 2).

Table 4 shows that 37,163 out of 211,611 deaths (17.6%) due to GC-COVID-related were redistributed to COVID-19 cases. After redistribution, we estimated an increase of 18.0% nationally in the total of COVID-19 deaths registered in 2020, higher in the elderly (Table 4). These proportions were higher in the interior of the country than in capitals (S2 Table).

**Table 4:** GC-COVID-related deaths redistributed as COVID-19 cases according to age. Brazil, 2020.

Age-group	Total deaths	Total GC-COVID-related	Redist-GC-COVID-related	COVID-19 reported	COVID-19 estimated	% increase
0–59	462,344	51,836	7105	47764	54869	14.9
60 +	1,066,851	159,775	30058	158860	188918	18.9
Total *	1,529,195	211,611	37,163	206,624	243,787	18.0

* Missing age not included.

## Discussion

Our research provides a first look into the undercount of COVID due to misclassification among garbage codes. It indicates that official statistics on COVID mortality were underestimated in the three selected capitals. Between February and June 2020, we found that COVID-19 was in fact the underlying cause of death in 23.4% of deaths registered as GC-COVID-related cases (severe acute respiratory syndrome, pneumonia unspecified, sepsis, respiratory failure and ill-defined causes). These results are in line with other studies, which have already pointed out the issue of underreporting of deaths from COVID-19 in Brazil and other countries [15–17].

Our study is the first ever systematic population-based study carried out in hospitals and health facilities for detecting under registration of COVID-19 among registered GCs in mortality statistics. The fact that deaths with GC-COVID-related cases are hidden COVID-19 deaths during the pandemic period is not surprising. If we consider that these causes were responsible for 211,611 deaths in Brazil in 2020, using simple mathematics, we could infer an increase of 18% in the number of deaths from COVID-19 officially registered in the mortality information system.

In 2020, there was an increase in the incorrect classification of causes of death with the worsening in quality of medical certification due to the overload of services and health professionals, impacting the increase of garbage codes. This is explicit when comparing data for the years 2017–2019. There was an increase in causes of death coded as sepsis (+ 30%), ICD-10 Chapter XVIII ill-defined causes (+ 39%), respiratory failure (50%), SARS (381%), etc. However, unspecified pneumonia as the cause of death dropped by 16%, possibly because the flu virus circulated less in 2020 due to the predominance of the SARS-COV-2 virus, and decreased social mobility. It is most probable that many physicians certified SARS as a cause of death rather than unspecified pneumonia in most cases of COVID-19 disease.

We believe that the underestimation of COVID-19 mortality of 18% is likely its lower bound. This conclusion is based on the proportions of COVID-19 deaths detected among some causes (GC-COVID-related causes) registered in vital statistics as underlying causes of death in the first 5 months of the epidemic in Brazil. In fact, if we had used the proportions found in May-June the underestimation would be higher. In addition, we have investigated only a few causes which could impact COVID-19 death numbers. For example, some other causes such as chronic obstructive pulmonary disease, which is a contributing condition of COVID-19 [11], could also have hidden this disease. Also, under notified deaths in the SIM were not taken into account. This most probably occurred when severely ill individuals were hospitalized in underprivileged municipalities of the North and Northeast regions, as the mortality is related with socioeconomic characteristics such as geographic location of the hospital [18]. Our findings of higher number of misclassified COVID-19 in the interior of the country, which is in line with the study cited above, is probably due to less access to health assistance and diagnostic resources such as laboratory tests. Many cities in the interior of the country have no intensive care units (ICUs) and patients are sent to other cities, with corresponding delays in treatment due to the collapse of public health system with the progression of the epidemic [19]. Finally, the validity of our statement that the true underestimation of COVID-19 deaths is certainly larger than our findings, is confirmed by another publication on this topic, which estimates a higher underreporting of COVID-19 mortality (23%) using various national data sources and statistical methods for comparing COVID-19 deaths, SARS and natural causes of death [20].

It is standard practice in burden of disease studies to redistribute garbage codes to other probable causes of death based on algorithms for each age-sex-location-year GC group registered. Multiple cause analysis, negative correlation, and proportional redistribution are the three most frequent methods used for defining the target codes to which deaths are redistributed and the redistribution factors [21]. Concerning the redistribution method of GC-COVID-related deaths adopted in our study the rationale for this is that it was based on hospital investigations of these causes, probably more adequate for correcting COVID-19 misclassification. We used resulting proportions of COVID-19 deaths detected from the investigation process as inputs to redistribute a fraction of GC-COVID-related cases by age groups, cause-specific and location. Given that there could be other non-COVID-19 causes among unspecified pneumonia, sepsis, respiratory failure or ill-defined causes, only a fraction of deaths due to these cases (17.6%) was attributed to COVID-19 deaths in this study.

The high burden of GC-COVID-related deaths in 2020 probably reflects uneven testing availability in the country due to the national health policy fragmentation with discontinued funding and distribution of tests [22] which could cause differences in diagnostic capacity and therefore in death counts [23]. The lack of tests for the population was higher in the first months of 2020, and over time testing has increased [6,19]. However, even when tests are available, there are quality problems in the collection of clinical specimens for specific diagnosis, with false-negative results due to the inappropriate period of collection or the quality of packaging and transport of the material to the laboratories [24]. Experiences from many countries that do not test enough show that this is reflected in the quality of the monitoring indicators of COVID-19 [25].

The higher detection of COVID-19 among GC-COVID-related with the progression of the epidemic may be the result of the availability of more laboratory tests to confirm the diagnosis in the investigation process. Unfortunately, patients sometimes died before results were available and this information may not have been timely enough to help the certifying physician assign the correct cause of death. Therefore, efforts should be directed towards better quality of medical care and more access to quality health services [26], and also in training of physicians regarding death certificate completion [27], particularly in the dramatic epidemic situation in the country in which the counting of deaths is of great interest.

## Strengths and limitations of study

The interpretation of our results depends on two premises. The first one is that investigation by trained personnel using a standardized questionnaire conducted in hospitals and other health facilities for selected deaths with COVID-related garbage codes yields more valid information regarding the true underlying cause of death than that on their death certificates. One important strength of this study is that our initiative has been built on prior hospital and home investigations of garbage codes focused on ill-defined causes since 2006 [26] and, more recently in several other GCs, for example sepsis [28]. After the investigation, the specific causes detected in the study actually replaced the original codes for the underlying cause of death in the final released data of official vital statistics. The results indicate the applicability of the investigation of GCs in order to correct misclassification in the mortality information system through mortality surveillance [5].

The second premise is that the three cities studied can be considered a portrait of Brazil and that, therefore, the results obtained for them can be inferred for Brazil as a whole. This premise is not easily proven in a country as large and diverse as Brazil. Our intention, when making the inference of the results, was more to alert to the possibility that there is misclassification in the country and that it may represent a large proportion of deaths than specifically to establish a precise estimate for this proportion. However, the increase in COVID mortality that we estimated is likely to be an underestimation of the overall COVID excess mortality during the pandemic given the fact that we have not investigated all possible deaths which could be due to COVID. In fact, for the few deaths that we investigated which did not have COVID-related GC as the underlying cause but that had some mention of COVID on any line of the death certificate, we did detect a high proportion of COVID-19. These deaths were not considered in the redistribution method in this study, because they were not systematically included. Besides, although the probability of COVID-19 as underlying cause of death among deaths registered in the selected municipalities as GC-COVID-related was higher in May-Jun compared to Feb-Apr, we had applied an average of these proportions for extrapolating the study results to national GC-COVID-related deaths. Probably it would have been more appropriate to have applied the proportions of May-June, but we preferred a more conservative option, and most probably we may have under stated the likely undercount because the second semester probably followed the pattern found in May-June.

Additional limitations include our inability to identify the cause of death among ill-defined cases due to difficulties in accessing adequate information. The lowest redistribution factors for these causes applied in states (1.7% for the age group 0–59 years and 5.1% used for mortality in ages 60 years and over) were probably an underestimation of the true redistribution factors and probably underscore the states with higher proportions of ill-defined causes. On the other hand, states with higher proportions of SARS and pneumonia, which were in some sense more indicatives of COVID-19 than the ill-defined causes, were more weighted in the final state redistribution result.

Another potential limitation of our analysis concerns the diagnostic criteria for COVID deaths. Although the evidence criteria have considered clinical aspects, the majority of cases had laboratory confirmation, in particular after April, probably due to better access to PCR exams for severe cases. In addition, the clinical picture was sufficient for confirmation as data collected for suspected COVID-19 indicates that 94% were in fact this disease.

Concerning the issue of completeness of total deaths, considering SIM in this study as the source of cases to be investigated has the advantage that this system probably has better quality in monitoring deaths than the surveillance system SIVEP-Gripe, which is based on passive notifications of hospital admissions due to SARS, and contains fewer COVID-19 deaths if compared with the SIM database [29]. Although timely reporting remains a challenge in the present context, the pandemic has highlighted the enormous value of the SIM and the need to improve its utility in detecting and monitoring the mortality burden of emerging diseases.

## Conclusions

The amount of misclassification of COVID-19 mortality occurring in health facilities in three capitals of Brazil was relevant. Almost a quarter of GC-COVID related deaths investigated were in fact COVID-19 cases. This scenario has implications for vital statistics in the country as we have an important number of GC-COVID-cases reported in each state which could hide this disease. So, we estimate that the burden of COVID-19 disease was probably under estimated at least by 18% in the country in 2020.

Information is vital for public health policy and having the ability to assess the quality of data is essential for extracting valid information that will guide public health actions. Stakeholders in Brazilian cities and states should know more about misclassification within their own data, and possibilities of bettering estimates of their own COVID-19 loads. Applied surveys carried out in partnership with stakeholders, such as this, provide useful information for health analysis and decision-making support. This research confirms the importance and the opportunity for mortality surveillance of deaths due to GCs and calls for specific attention to the importance of availability of laboratory exams and medical training. Therefore, initiatives should be established to expand testing capacity at SUS (Unified Health System), which is also a strategy to reduce and support GC-COVID-related investigations.

## Supporting information

S1 Table Other underlying causes not GC-COVID-related investigated: HIV/AIDS, Neoplasms, Stroke, Ischemic heart disease, Other cardiovascular and circulatory diseases, Chronic obstructive pulmonary disease, Other chronic respiratory diseases, Other respiratory diseases, Diabetes mellitus, Chronic kidney disease, Other causes. (XLSX)

S2 Table Capital: All deaths occurring in 27 capitals of the country; Interior: All deaths occurring in the country, except those in capitals. (XLSX)

S1 Data Municipality: Belo Horizonte = Belo Horizonte; Natal (residentes) = Natal; Natal (ocorrencia)., Residente outros municípios = deaths in Natal from other municipalities; Salvador = Salvador, Sex: Feminino = Female; Masculino = Male, Age: 60 e mais = 60 and more; 30 a 59 anos = 30 to 59 years; 0 a 29 anos = 0 to 29 years, Month of death: month, Original cause-of-death: CMD = ill-defined cause of death; SRAG = SARS; Pneumonia = unspecified pneumonia; Septicemia = sepsis; I_RESP_AG = respiratory failure. Diagnostic confirmation criteria: Definitivo = COVID- definitive criterion; Provável: COVID- probable criterion; Possível: COVID- possible criterion; Another diagnosis not related to COVID-19: Another specific diagnosis not COVID-19; Lack of information: Undetermined underlying cause. Final cause-of-death: COVID, Other causes non COVID.
(XLSX)
